# Defining the Efficacy and Safety of Phosphodiesterase Type 5 Inhibitors with Tamsulosin for the Treatment of Lower Urinary Tract Symptoms Secondary to Benign Prostatic Hyperplasia with or without Erectile Dysfunction: A Network Meta-Analysis

**DOI:** 10.1155/2020/1419520

**Published:** 2020-03-26

**Authors:** Chengquan Ma, Jianzhong Zhang, Zhonglin Cai, Jian Xiong, Hongjun Li

**Affiliations:** Department of Urology, Peking Union Medical College Hospital, Peking Union Medical College, Chinese Academy of Medical Sciences, No 1. Shuaifuyuan Beijing, China

## Abstract

**Purpose:**

The purpose of this study was to compare the relative safety and efficacy of different types of phosphodiesterase type 5 inhibitors (PDE5-Is) with tamsulosin for the treatment of lower urinary tract symptoms (LUTS) secondary to benign prostate hyperplasia (BPH) (BPH-LUTS) with or without erectile dysfunction (ED).

**Methods:**

We use the Stata version 13.0 to conduct the network meta-analysis (NMA) with a random effects model of the Bayesian framework. The International Prostate Symptom Score (IPSS), Maximum Urinary Flow Fate (*Q*_max_), International Index of Erectile Function (IIEF), and their credible intervals (CI) were used to compare the efficacy and safety of every medical intervention, including sildenafil plus tamsulosin, tadalafil plus tamsulosin, and vardenafil plus tamsulosin.

**Results:**

Seven RCTs including 531 participants with seven interventions were analyzed. The results of NMA SUCRA showed that compared with different doses or types of PDE5-Is combined with tamsulosin (0.4 mg qd), the sildenafil (25 mg qd) combined with tamsulosin (0.4 mg qd) group had the greatest probabilities of being the best in the achievement of improving IIEF. The sildenafil (25 mg 4 days per week) combined with tamsulosin (0.4 mg qd) group had the greatest probabilities of being the best in the achievement of improving *Q*_max_, whereas sildenafil (25 mg qd) combined with tamsulosin (0.4 mg qd) ranked the best for the safety outcomes.

**Conclusions:**

This meta-analysis indicates that sildenafil combined with tamsulosin is the best effective and tolerated treatment option for BPH-LUTS with or without ED. Further RCTs are strongly required to provide more direct evidence.

## 1. Introduction

Recently, BPH-LUTS and ED had both been found to be highly prevalent conditions in elderly men and are usually becoming evident with the aging growth [1, 2]. Moreover, BPH-LUTS has been regarded as an independent risk factor for ED in elderly men and 94% of patients with severe LUTS having coexistent ED [3]. Treatment options for BPH-LUTS included *α*1-adrenoceptor antagonists (*α*1-blockers—tamsulosin, alfuzosin, and doxazosin) proposed as the first-line drug. The therapeutic drug for ED including oral PDE5-Is (such as tadalafil, sildenafil, mirodenafil, vardenafil, and udenafil) was also found recently to treat BPH-LUTS effectively. An oral drug to treat both conditions (BPH-LUTS and ED) is a major method though the therapeutic strategy is still not clear enough up to now. Further studies should focus on the treatments for BPH-LUTS with or without ED.

There are two articles that conducted a systematic review and meta-analysis concerning the use of PDE5-Is in BPH-LUTS, finding that PDE5-Is can significantly improve LUTS and erectile dysfunction treatment [4, 5]. Subsequently, several meta-analyses have defined the efficacy and safety of PDE5-Is alone or in combination with *α*1-blockers for the treatment of BPH-LUTS with or without ED [6, 7]. And the combination therapy can significantly improve IPSS, *Q*_max_, and IIEF; it might be more suitable for patients with BPH-LUTS with or without ED [7]. However, all these studies were used just to compare the efficacy and safety of combination therapy (*α*1-blockers plus PDE5-Is) with monotherapy (*α*1-blockers or PDE5-Is alone); there was no study to compare the efficacy and safety of different combined arms (such as sildenafil plus tamsulosin versus vardenafil plus tamsulosin versus tadalafil plus tamsulosin) for treating BPH-LUTS with or without ED. Therefore, we conducted a NMA to define the best candidates to improve LUTS and erectile dysfunction treatment by assessing IPSS, *Q*_max_, and IIEF changes.

## 2. Methods

This study was registered with PROSPERO (number CRD 42019139062), and we used the same research methods, the study protocol that had been published by our team on January 2020 [8]. The 25 items reported determines the score of every RCT in Table 1.

## 3. Results

### 3.1. Baseline Characteristics of Included Studies

This NMA initially retrieved a total of 118 related literatures and 7 RCTs which satisfied the inclusion criteria and were finally included in this NMA to compare the efficacy and safety of eight regimens. The baseline characteristics of each trial are presented in Table 2.

**Figure 1:**
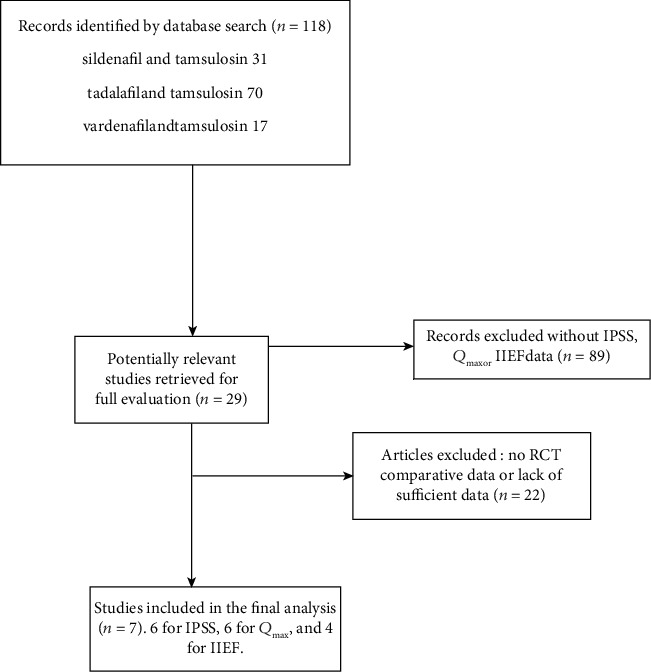
Flow diagram of this network meta-analysis.

Among the 7 studies, six trials were used to compare the relative IPSS's improving efficacy of different kinds of PDE5-Is with tamsulosin for the treatment of BPH-LUTS with or without ED [6, 9–13]; six trials were used to compare the relative *Q*_max_'s improving efficacy [6, 10–14]; four trials were used to compare the relative IIEF's improving efficacy [6, 10, 11, 13], and six trials were used to compare the relative safety [6, 9–13] (Figure 1). The ranking of probability of different interventions was estimated by comparing the SUCRA shown in Table 3.

### 3.2. IPSS and IIEF Changes

**Figure 2:**
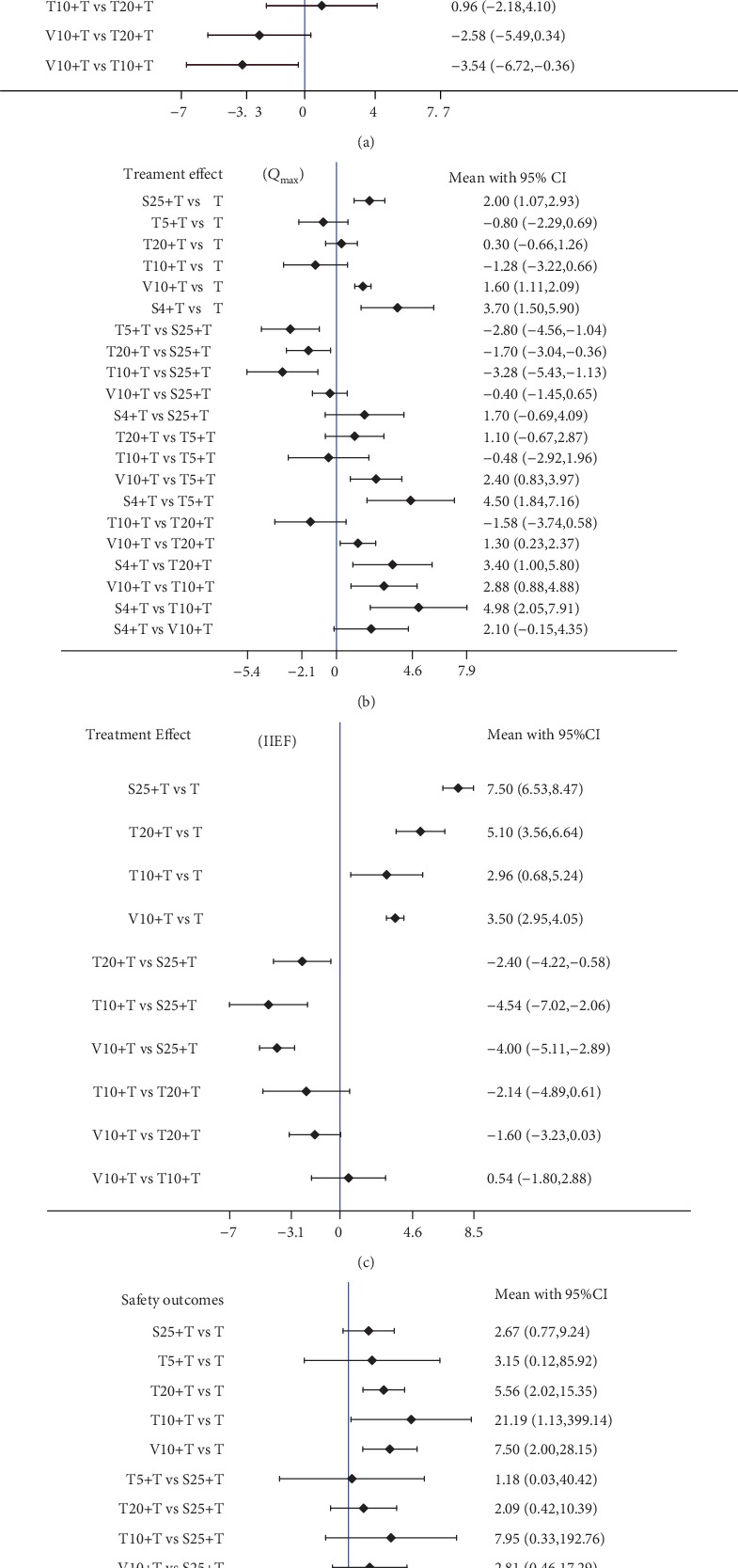
Network forest plot of treatment comparisons for efficacy and safety. (a) The IPSS of treatment comparisons. (b) The *Q*_max_ of treatment comparisons. (c) The IIEF of treatment comparisons. (d) The safety outcomes of treatment comparisons. T: tamsulosin (0.4 mg qd); S25+T: sildenafil (25 mg qd) plus tamsulosin (0.4 mg qd); T20+T: tadalafil (20 mg qd) plus tamsulosin (0.4 mg qd); V10+T: vardenafil (10 mg qd) plus tamsulosin (0.4 mg qd); T10+T: tadalafil (10 mg qd) plus tamsulosin (0.4 mg qd); T5+T: tadalafil (5 mg qd) plus tamsulosin (0.4 mg qd); and S4+T: sildenafil (25 mg 4 days/week) plus tamsulosin (0.4 mg qd).

Sildenafil (25 mg qd) combined with tamsulosin (0.4 mg qd) is listed on top of the league table, because it was associated with the most favorable SUCRA for the IPSS and IIEF changes. The results indicated that compared with sildenafil with tamsulosin, tadalafil with tamsulosin, and vardenafil with tamsulosin, sildenafil (sildenafil 25 mg qd) combined with tamsulosin (0.4 mg qd) can greatly improve the efficacy of treatment for BPH-LUTS with or without ED. When considering IPSS, compared with sildenafil (25 mg qd) combined with tamsulosin, vardenafil (10 mg qd) combined with tamsulosin was ranked second. However, compared with sildenafil (25 mg qd) combined with tamsulosin, tadalafil (20 mg qd) combined with tamsulosin was ranked second for improving IIEF efficacy (Figures 2(a) and 2(c).

### 3.3. *Q*_max_ Improving

The sildenafil (25 mg 4 days per week) combined with tamsulosin (0.4 mg qd) group had the greatest probabilities of being the best in the achievement of improving *Q*_max_, while sildenafil (25 mg qd) combined with tamsulosin (0.4 mg qd) ranked second in the assessment of improving *Q*_max_. The results indicated that compared with sildenafil with tamsulosin, tadalafil with tamsulosin, and vardenafil with tamsulosin, sildenafil (25 mg 4 days per week) combined with tamsulosin (0.4 mg qd) group can greatly improve the efficacy of treatment for BPH-LUTS with or without ED (Figure 2(b)).

### 3.4. The Safety Outcomes

The sildenafil (25 mg qd) combined with tamsulosin (0.4 mg qd) group had the greatest probabilities of being the least in the achievement of adverse events. The results indicated that compared with tadalafil with tamsulosin and vardenafil with tamsulosin, the sildenafil with tamsulosin group has the greatest probabilities of having the best tolerability treatment for BPH-LUTS with or without ED (Figure 2(d)).

## 4. Discussion

This is the first article to prospectively assess the effects and safety of different types of PDE5-Is with tamsulosin combination therapy on subdomains of BPH having LUTS with or without sexual function in men. We estimated the treatment effects and tolerability of different combined interventions based on the NMA method according to the indirect evidence from 7 RCTs in patient. Then we know that the sildenafil (25 mg qd) combined with tamsulosin is the best choice to improve the efficacy of IPSS and IIEF treatment for BPH-LUTS with or without ED symptoms. And sildenafil (25 mg 4 days/week) combined with tamsulosin proved superior to both sildenafil (25 mg qd)+tamsulosin and to all other combined interventions to improve the efficacy of *Q*_max_. The tadalafil (10 mg/day) combined with tamsulosin has the best tolerability than other combined groups.

The potential negative impact of *α*1-blockers and/or 5*α*-reductase inhibitors, especially the side effect on sexual function for young patients, for treating BPH-LUTS may be the barrier for clinicians to prescribe these drugs [15]. It is worth noting that the reported articles about adverse events of the combination treatment were based only on incidence. Thus, mentioning about the adverse events during follow-up may be biased due to patients' symptom misinterpretation and nonquantitative propensity. Compared with untreated hypogonadal men, long-term testosterone-treated hypogonadal men can significantly improve urine and sexual function [16]. And it was found that dutasteride increased the severity of erectile dysfunction in the treatment of BPH [17]. In light of this, Traish AM et al. revealed that finasteride aggravates the symptoms of ED and could decrease the testosterone levels for patients with BPH; however, they could not find the side effect for tamsulosin [18]. And the *α*1-blockers have been investigated potential therapeutic for ED [19]. Therefore, *α*1-blockers (tamsulosin) can be useful and safe to treat BPH-LUTS with or without ED.

*α*1-blockers were widely prescribed drugs, and PDE5-Is had been becoming popular recently for treating BPH-LUTS. For the legal sense, the PDE5-Is got an approval in the USA recently for the treatment of BPH-LUTS with or without ED. As we all know, the main mechanisms of PDE5-Is are playing an important role in the nitric oxide (NO) pathway to relax the smooth muscle. NO mediates relaxation for the corpus cavernosum muscle and bladder. PDE5-Is lead to increase NO in the smooth muscle, stimulating penile erection and prostate or bladder neck and blood vessel relaxation [20–23]. These were the mechanisms of PDE5-Is to increase the penile blood flow and to induce improvement of treating ED and BPH-LUTS. Other mechanisms in studies of PDE5-Is in humans have been conducted that increase the Rho-kinase activity, enhance afferent nerve activity and excessive autonomic nervous system, and revert fibroblast-to-myofibroblast transdifferentiation, which might affect the clinical results of PDE5-Is for LUTS [24–28]. That is why PDE5-Is get extensive attention for treating BPH-LUTS currently.

PDE5-Is can induce relaxation of urethra's smooth muscle cells similar to the promising targets for drug use for the urogenital tract. Firstly, while PDE5 is expressed in several organs including the prostate [29], bladder [30, 31], vascular smooth muscle [32], testis [33], and corpus cavernosum [34], the PDE6 isoform is mainly expressed in the eyes of mammals which are the primary visual transduction effectors in cones and rods [35, 36]. It is well known that first-generation PDE5 inhibitors, such as sildenafil and vardenafil, are also capable of inhibiting PDE6 subtype. The reason was the structure similar to PDE5 and PDE6 but have functional differences [37]. Secondly, the PDE5/PDE6 ratio of tadalafil can have high selectivity than other kinds of PDE5-Is such as sildenafil or vardenafil [38]. And the visual function could be the important side effect after the inhibition of PDE6 [39]. Thirdly, the special pharmacokinetic properties of tadalafil have better selectivity for PDE5 compared to PDE6, as tadalafil may have the potential to change the systemic exposure through CYP3A4 metabolic pathway [40]. Fourthly, the neurogenic contractions of the peripheral prostate and bladder neck could be inhibited by tamsulosin, and tadalafil can enhance this inhibitory effect to perform more excellent efficacy on sexual and voiding dysfunction [20]. The combination of tadalafil plus tamsulosin is widely used to treat ED and BPH-LUTS. That is why tadalafil is well tolerated and efficacious to treat LUTS associated with both BPH and ED [41, 42]. However, this is inconsistent with our findings that sildenafil combined with tamsulosin is the best well-tolerated and efficacious intervention for patients. We consider the reason that may be less RCTs included were analyzed, which could be the source of bias. Therefore, a direct comparison of various medical interventions is required to confirm these effects.

Several limitations of our analysis should also be stressed. Because of a lack of direct head-to-head trials, researchers often depended on an NMA analytic tool to examine the comparative effectiveness or safety of drugs to cure patients. And NMA can include lots of evidence to rank order every intervention by calculating the relative effectiveness. We graded indirect treatment comparison analyses as low strength of evidence. This method is a surrogate for direct comparison though it had been confirmed to be validated for the NMA of RCT comparing outcomes. Further RCTs are recommended to test our NMA results and compare the effects directly from the different types of PDE5-Is plus tamsulosin. Additionally, different dosages of PDE5-Is in combined arms can be conducted by subgroup analyses. Another limitation of this study is the difference in the duration of treatment among the 7 RCTs included, which may affect the final results.

In conclusion, while indirect comparisons cannot supplant direct comparative data, we performed a Bayesian NMA including 7 RCTs and finally found that sildenafil combined with tamsulosin is the best efficacious intervention in treating LUTS and improving IIEF for patients, and it is also well tolerated than other combinations. Defining the best combined therapy candidates based on clinical features and the severity of LUTS can provide clinically relevant benefits for patients.

## Figures and Tables

**Table 1:** 25-item CONSORT checklist.

Study	1	2	3	4	5	6	7	8	9	10	11	12	13	14	15	16	17	18	19	20	21	22	23	24	25	Total score
Karami 2016	✓	✓	✓	✓	✓	✓	×	✓	×	✓	×	✓	✓	✓	✓	✓	✓	✓	✓	✓	✓	✓	×	✓	✓	21
Fawzi 2016	✓	✓	✓	✓	✓	✓	✓	✓	✓	✓	✓	✓	✓	✓	✓	✓	✓	✓	✓	✓	✓	✓	×	✓	✓	24
Singh 2014	✓	✓	✓	✓	✓	✓	×	×	×	✓	×	✓	✓	✓	✓	✓	✓	✓	✓	✓	✓	✓	×	✓	✓	20
Regadas 2012	✓	✓	✓	✓	✓	✓	×	×	×	✓	×	✓	✓	✓	✓	✓	✓	✓	✓	✓	✓	✓	×	✓	✓	20
Gacci 2012	✓	✓	✓	✓	✓	✓	×	✓	✓	✓	✓	✓	✓	✓	✓	✓	✓	✓	✓	✓	✓	✓	×	✓	✓	23
Tuncel 2009	×	✓	✓	✓	✓	✓	×	✓	×	✓	×	✓	✓	✓	✓	✓	✓	✓	×	×	✓	✓	×	✓	×	17
Bechara 2008	×	✓	✓	✓	✓	✓	×	✓	✓	✓	✓	✓	✓	✓	✓	✓	✓	✓	✓	✓	✓	✓	×	✓	×	21

1-25 indicates the specific items in the CONSORT checklist. ✓: fulfilled the item, ×: did not fulfill the item.

**Table 2:** Characteristics of individual studies included in the Network meta-analysis.

Author ID/year	Country	*N* (C/T)	Mean age	Treatment duration (months)	C: combined therapy T: tamsulosin or placebo+tamsulosin	Total IPSS (C/T)	*Q*_max_ (C/T)	IIEF (C/T)
Karami 2016	Iran	58/59	68.2	3	C: tadalafil (20 mg qd)+tamsulosin (0.4 mg qd) T: tamsulosin (0.4 mg qd)	10.1 ± 3.2/10.6 ± 3.5	15.9 ± 2.1/15.6 ± 3.1	17.2 ± 3.2/12.1 ± 5.1
Fawzi 2016	Egypt	63/68	66.0	6	C: sildenafil (25 mg qd)+tamsulosin (0.4 mg qd) T: placebo+tamsulosin (0.4 mg qd)	13.1 ± 4.5/17.6 ± 4.1	14.9 ± 3/12.9 ± 2.4	22.9 ± 2.3/15.4 ± 3.3
Singh 2014	India	44/45	62	3	C: tadalafil (10 mg qd)+tamsulosin (0.4 mg qd) T: tamsulosin (0.4 mg qd)	10 ± 2.989/10.26 ± 3.218	12.26 ± 3.537/13.54 ± 5.587	17 ± 5.705/14.04 ± 5.254
Regadas 2012	Brazil	20/20	60.4	1	C: tadalafil (5 mg qd)+tamsulosin (0.4 mg qd) T: placebo+tamsulosin (0.4 mg qd)	10.9 ± 5.1/14.4 ± 3.6	5.2 ± 2.4/6.0 ± 2.4	NM
Gacci 2012	Italy	30/30	68.0	3	C: vardenafil (10 mg qd)+tamsulosin (0.4 mg qd) T: placebo+tamsulosin (0.4 mg qd)	12.9 ± 1.0/16.7 ± 1.1	12.1 ± 1.1/10.5 ± 0.8	19.4 ± 0.8/15.9 ± 1.3
Tuncel 2009	Turkey	20/20	58.8	2	C: sildenafil (25 mg 4 days/week)+tamsulosin (0.4 mg qd) T: tamsulosin (0.4 mg qd)	NM	20.0 ± 3.6/16.3 ± 3.5	NM
Bechara 2008	Argentina	27/27	63.7	3	C: tadalafil (20 mg qd)+tamsulosin (0.4 mg qd) T: placebo+tamsulosin (0.4 mg qd)	10.2 ± 3.8/12.7 ± 5.1	NM	NM

C/T: combined therapy versus tamsulosin; NM: not mentioned.

**Table 3:** The ranking of probability of different interventions was estimated by comparing the SUCRA.

Treatment	SUCRA	Pr best	Mean rank
For IPSS			
T	11.2	0.0	5.4
S25+T	86.3	51.7	1.7
T5+T	71.0	25.0	2.5
T20+T	36.2	0.3	4.2
T10+T	18.9	0.0	5.1
V10+T	76.6	23.0	2.2
For *Q*_max_			
T	33.8	0.0	5.0
S25+T	80.7	7.5	2.2
T5+T	15.2	0.0	6.1
T20+T	42.7	0.0	4.4
T10+T	8.8	0.0	6.5
V10+T	70.9	1.1	2.7
S4+T	98.0	91.4	1.1
For IIEF			
T	0.1	0.0	5.0
S25+T	99.9	99.6	1.0
T20+T	72.8	0.4	2.1
T10+T	35.0	0.0	3.6
V10+T	42.2	0.0	3.3
The safety outcomes of treatment comparisons			
T	93.2	68.7	1.3
S25+T	63.7	4.8	2.8
T5+T	56.0	24.3	3.2
T20+T	40.0	0.0	4.0
T10+T	16.3	2.1	5.2
V10+T	30.7	0.1	4.5
